# Protein Kinase CK2 Contributes to Glucose Homeostasis by Targeting Fructose-1,6-Bisphosphatase 1

**DOI:** 10.3390/ijms24010428

**Published:** 2022-12-27

**Authors:** Mandy Pack, Tim Nikolai Gulde, Michelle Victoria Völcker, Anne S. Boewe, Selina Wrublewsky, Emmanuel Ampofo, Mathias Montenarh, Claudia Götz

**Affiliations:** 1 Medical Biochemistry and Molecular Biology, Saarland University, Building 44, 66421 Homburg, Germany; 2 Institute for Clinical and Experimental Surgery, Saarland University, Building 65, 66421 Homburg, Germany

**Keywords:** protein kinase CK2, fructose-1,6-bisphosphatase, gluconeogenesis, diabetes-associated genes

## Abstract

Glucose homeostasis is of critical importance for the survival of organisms. It is under hormonal control and often coordinated by the action of kinases and phosphatases. We have previously shown that CK2 regulates insulin production and secretion in pancreatic β-cells. In order to shed more light on the CK2-regulated network of glucose homeostasis, in the present study, a qRT-PCR array was carried out with 84 diabetes-associated genes. After inhibition of CK2, fructose-1,6-bisphosphatase 1 (FBP1) showed a significant lower gene expression. Moreover, FBP1 activity was down-regulated. Being a central enzyme of gluconeogenesis, the secretion of glucose was decreased as well. Thus, FBP1 is a new factor in the CK2-regulated network implicated in carbohydrate metabolism control.

## 1. Introduction

The pancreas is a vital glandular organ of vertebrates which is composed of an exocrine part and an endocrine part [1]. The smaller endocrine tissue of the pancreas contains the islets of Langerhans, which are homogeneously distributed across the pancreas and which enter into the exocrine glandular tissue. They are composed of five cell types, which produce different hormones [2]. The largest proportion of the islets of Langerhans are the β-cells (60–80%), which secrete insulin, followed by the glucagon-producing α- cells with 10–20% [2]. The main task of the α- and β-cells of the endocrine pancreas is the maintenance of glucose homeostasis, which is ensured by the secretion of the hormone insulin at high glucose levels in the blood and its antagonist, glucagon, at low glucose levels.

For the proper function of the β-cell tasks, and thus for the correct regulation of glucose-stimulated insulin secretion, a number of hormones and transcription factors play important roles. Among the most important transcription factors of the β-cell is the homeodomain transcription factor PDX1 (Pancreatic Duodenal Homeobox 1), also known as Idx1, Ipf1, Stf1 or IUF1, which is important for organ development as well as β-cell differentiation and the maintenance of the β-cell function [3]. The most important way of regulation of enzymes and transcription factors is phosphorylation and dephosphorylation, which is performed by protein kinases and protein phosphatases [4].

Among these protein kinases is CK2, which is already known to be implicated in the regulation of the carbohydrate metabolism and in particular in insulin production in β-cells of the pancreas [5,6,7]. This highly conserved and ubiquitously expressed protein kinase was first identified in 1954 by Burnett and Kennedy [8]. CK2 is a serine/threonine kinase which is known to phosphorylate more than 500 different substrates in a cell [9], which shows the importance of CK2 for many cellular processes (for review see: [6,10,11,12,13,14,15,16,17]). The CK2 holoenzyme consists of two catalytic subunits (CK2α and/or CK2α’) and two regulatory CK2β subunits [18]. CK2 phosphorylates threonine 231 and serine 232 in the polypeptide chain and thereby regulates PDX1. Inhibition of CK2 leads to an elevated synthesis and secretion of insulin from pancreatic β-cells [19,20,21]. Recently, Ca_V_2.1, a Ca^2+^ ion channel in pancreatic β-cells, was identified as a CK2 substrate and that CK2 phosphorylation of Ca_V_2.1 regulates Ca^2+^ entry in pancreatic β-cells and thereby insulin secretion [22]. The pancreatic TRPM3 channel is another substrate of CK2, which is also responsible for an elevated Ca^2+^ entry into the cytoplasm after CK2 inhibition [23].

To identify further mechanisms by which CK2 may affect glucose metabolism, in the present study, we performed a qRT-PCR array with 84 diabetes-associated genes in pancreatic β cells. We found a down-regulation of the expression of fructose-1,6-bisphosphatase also known as FBP1 after inhibition of CK2 kinase activity. FBP1 is one of the two isoforms, the liver isoform FBP1 and the muscle isoform FBP2 [24]. FBP1 is a key enzyme in the gluconeogenesis pathway, which is mainly localized in the liver. It catalyzes the hydrolysis of fructose-1,6-bisphosphate to fructose-6 phosphate and inorganic phosphate. We also found a down-regulation of FBP1 expression also in liver cells after pharmacological inhibition of CK2 kinase activity as well as down-regulation of CK2α and CK2α’ by siRNA technology. The down-regulation of FBP1 resulted in a reduced phosphatase activity, which finally led to the reduced secretion of glucose from liver cells. In an animal model we could confirm these results in vivo. Thus, these results indicate that CK2 is implicated in the glucometabolic control not only by regulating β-cell endocrine function as previously shown but also via direct regulation of one of the key enzymes of gluconeogenesis.

## 2. Results

**Figure 1:**
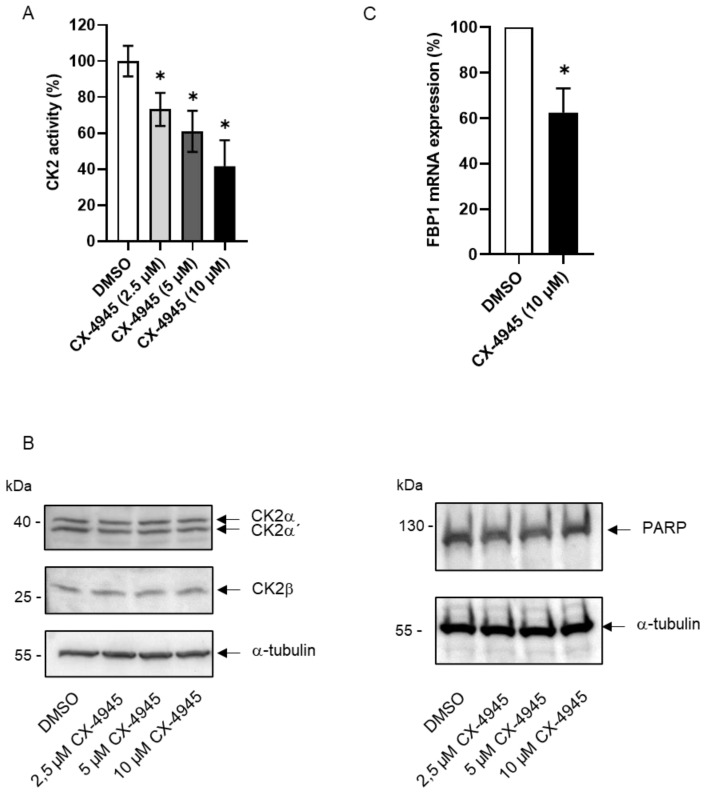
Inhibition of CK2 activity by CX-4945 in INS-1 832/13 cells. INS-1 832/13 cells were treated with 2.5, 5 or 10 µM CX-4945 or an equal volume of the solvent DMSO for control for 24 h. Cells were harvested, protein or RNA extracted and subjected to the following analyses. (**A**) An amount of 10 µg total protein was used for an in vitro phosphorylation with the CK2-specific substrate peptide RRRDDDSDDD. Incorporation of [^32^P]-labelled phosphate into the peptide in the presence of the inhibitor was set in reference to the amount of incorporated phosphate of the DMSO control (100%). (**B**) An amount of 30 or 60 µg (for PARP detection) of total protein was separated on a 12.5% or 7.5% (for PARP) SDS polyacrylamide gel and blotted onto a PVDF membrane. CK2 subunits, PARP and loading control tubulin were detected by specific antibodies and enhanced chemiluminescence. (**C**) RNA was reverse transcribed into cDNA, which was then subjected to a qPCR analysis using FBP1-specific primers. The message was normalized with actin as housekeeping gene. The graph shows the result of three independent experiments. * Statistical significance was accepted as *p* < 0.05.

In previous studies, we identified protein kinase CK2 as a negative regulator of pancreatic β-cell function, mostly by targeting the key transcription factor PDX1 [19,20,21]. In order to shed more light on CK2-dependent pancreatic networks, we used the cell line INS-1 832/13, which is a derivative of the insulinoma cell line INS-1 [25]. This cell line responds to a glucose stimulus with an elevated production and secretion of insulin. For inhibition of the protein kinase activity we used the well- established inhibitor CX-4945 [26] also known as Silmitasertib. In order to find the most effective concentration of CX-4945, we exposed INS-1 832/13 cells to 2.5, 5 and 10 µM CX-4945 as well as to the solvent DMSO for control for 24 h and determined CK2 kinase activity in the cell extract. As shown in Figure 1A, CK2 kinase activity decreased dose-dependent. In order to exclude that the reduction in CK2 kinase activity might be due to a reduction in the expression of CK2 subunits, we examined the amount of the CK2 subunits by immunoblot analysis of cell extracts after treatment of cells with CX-4945. As shown in Figure 1B, treatment of INS-1 832/13 cells with CX-4945 had no influence on the expression of CK2α, CK2α’ and CK2β. It is known that CX-4945 may induce apoptosis depending on the cell type used [27,28,29,30,31,32]. Therefore, we tested whether CX-4945 might also induce apoptosis in INS-1 832/13 cells. We analyzed PARP cleavage as one of the hallmarks of apoptosis. In order not to miss the presence of the apoptotic cleavage product, we loaded a higher amount of total protein on a second gel. As shown in Figure 1B, we only detected full-length PARP but no increase in cleavage product indicating that the concentration of CX-4945 used here, had no effect on the viability of INS-1 832/13 cells. Hence, we decided to use the concentration of 10 µM for all further experiments.

We next performed an RT² Profiler PCR Array (Qiagen, Hilden, Germany) with 84 different diabetes-associated genes for the detection of new target molecules of CK2. In three independent experiments, we consistently found a reduced expression of the fructose-1,6-bisphosphatase 1 gene (FBP1) in cells treated with CX-4945 by a factor of 3. To verify these results by a qRT-PCR experiment, where we used FBP1-specific primers, we incubated INS-1 832/13 cells in the presence or absence (solvent control) of 10 µM CX-4945 for 24 h. As housekeeping control, we determined the message for β-actin. As shown in Figure 1C, we repeatedly observed a marked reduction in the expression of the FBP1 gene compared to the DMSO control.

**Figure 2:**
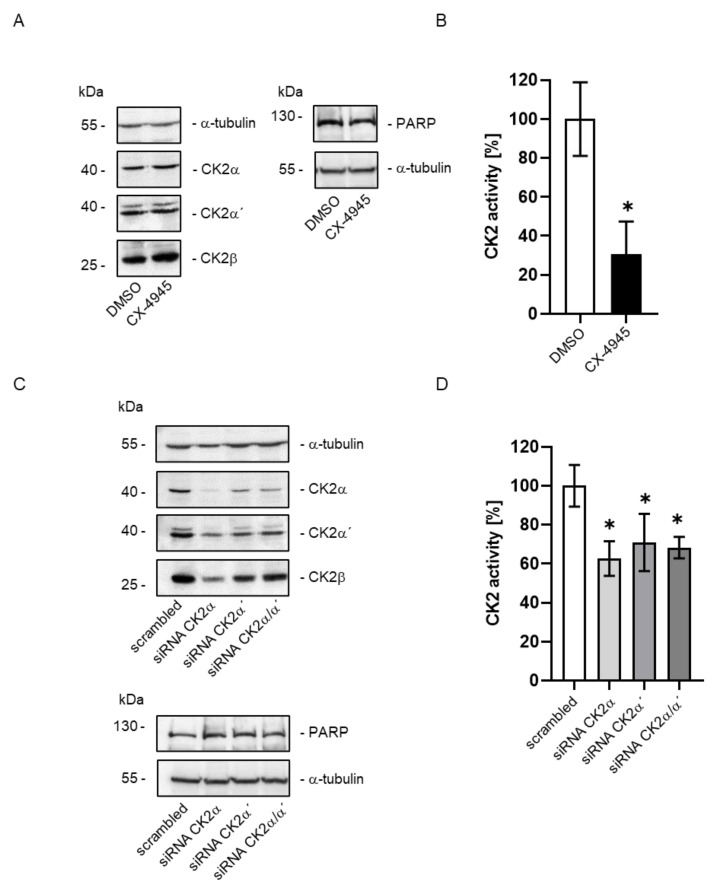
Down-regulation of CK2 expression or activity in McA-RH7777 cells. CK2 activity was either inhibited by treatment with 10 µM CX-4945 or an equal volume of DMSO (for control) or CK2 expression was down-regulated by transfection with siRNA against the catalytic subunits CK2α or CK2α´ (single or combined) or a scrambled siRNA for control. At 24 h after addition of CX-4945 or 72 h after transfection of the siRNA, cells were harvested and total protein extracted and subjected to the following analyses. (**A**,**C**) An amount of 30 or 60 µg (PARP) of total protein was separated on a 12.5% SDS polyacrylamide gel and blotted onto a PVDF membrane. CK2 subunits, PARP and loading control tubulin were detected by specific antibodies and enhanced chemiluminescence. One representative blot of at least 3 experiments is shown. (**B**,**D**) An amount of 10 µg total protein was used for an in vitro phosphorylation with the CK2-specific substrate peptide RRRDDDSDDD. Incorporation of [^32^P]-labelled phosphate into the peptide was set in reference to the amount of incorporated phosphate of the DMSO or the scrambled control (100%). * Statistical significance was accepted as *p* < 0.05.

To exclude that the effects observed above are cell type specific, we additionally exposed rat hepatoma cells McA-RH7777, which are known to highly express FBP1 [33,34]. Moreover, we down-regulated CK2 activity by two strategies: either we inhibited CK2 activity with CX-4945 for 24 h or we down-regulated CK2 by RNA interference targeting the catalytic subunits CK2α and CK2α’. Cells were harvested and analyzed 72 h after transfection. The control and treated cells of both experiments were harvested, and the cell extract analyzed by SDS gel electrophoresis followed by immunoblotting with CK2 subunit-specific antibodies. As shown in Figure 2, McA-RH7777 control cells (DMSO (Figure 2A) or cells transfected with scrambled siRNA (Figure 2C) expressed all three CK2 subunits. CX-4945 had no impact on the expression of the CK2 subunits (Figure 2A). Down-regulating the catalytic CK2 subunits by siRNA was successful in single transfection as well as in double transfection (Figure 2C). Treatment with CX-4945 or down-regulating the expression of one or both of the catalytic subunits should be reflected in changes in the intracellular CK2-activity. CK2 kinase activity was determined by using a CK2-specific substrate peptide in an in vitro phosphorylation assay. Residual activity in treated cells was referred to the activity of control cells (DMSO or cells treated with scrambled siRNA, respectively). Results are shown in Figure 2.

After an incubation of the cells with CX-4945 for 24 h, the CK2 kinase activity was reduced to approximately 35% compared to the solvent control (Figure 2B). CK2 activity in cells of the siRNA mediated down-regulation of CK2 was reduced to about 60% for CK2α and 65% for CK2α’ and the combination of both subunits (Figure 2D).

Since down-regulating CK2 activity and/or expression might have an impact on the viability of cells, we analyzed the apoptosis marker PARP for cleavage under those conditions. We observed neither in cells treated with CX-4945 (Figure 2A) nor in cells transfected with the different siRNA (Figure 2C) an increase in the apoptosis-related 89 kDa cleavage product of PARP. Thus, McA-RH7777 cells seem to be suitable for a subsequent qRT-PCR analysis for the analysis of expression of the FBP1 mRNA.

**Figure 3:**
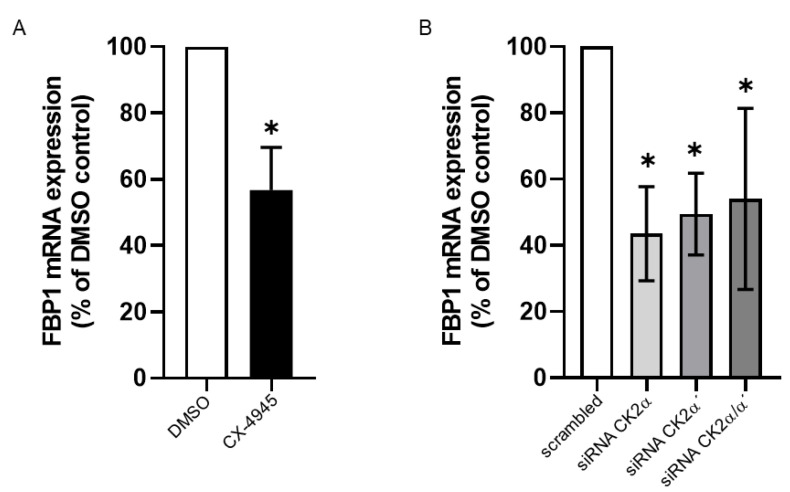
Impact of CK2 down-regulation on FBP1 mRNA expression. (**A**) McA-RH7777 cells were treated with 10 µM CX-4945 for 24 h. Control cells were incubated with an equal volume of the vehicle DMSO. (**B**) CK2 expression was down-regulated by transfection with siRNA against the catalytic subunits CK2α or CK2α´ (single or combined) or a scrambled siRNA for control for 72 h. Cells from A and B were harvested, and total RNA was isolated using the QIAzol lysis reagent. The mRNA amount of FBP1 was determined using qRT-PCR. After normalization to actin, the amount of FBP1 mRNA of control-treated cells was set 100% and the amount of treated mRNA cells calculated in reference to it. * Statistical significance was accepted as *p* < 0.05.

We treated McA-RH7777 cells either with CX-4945 or transfected them with siRNA against the CK2 subunits. As control, we used the solvent DMSO or scrambled nonsense siRNA for the transfection experiment. Cells were harvested, RNA extracted and subjected to qRT-PCR with primers for FBP1 and actin for normalization. Results are shown in Figure 3. In line with our results with INS-1 832/13 cells, we detected a substantial decrease in the mRNA level of FBP1 either after CX-4945 treatment (Figure 3A) or silencing the CK2α subunits with siRNA technology (Figure 3B).

**Figure 4:**
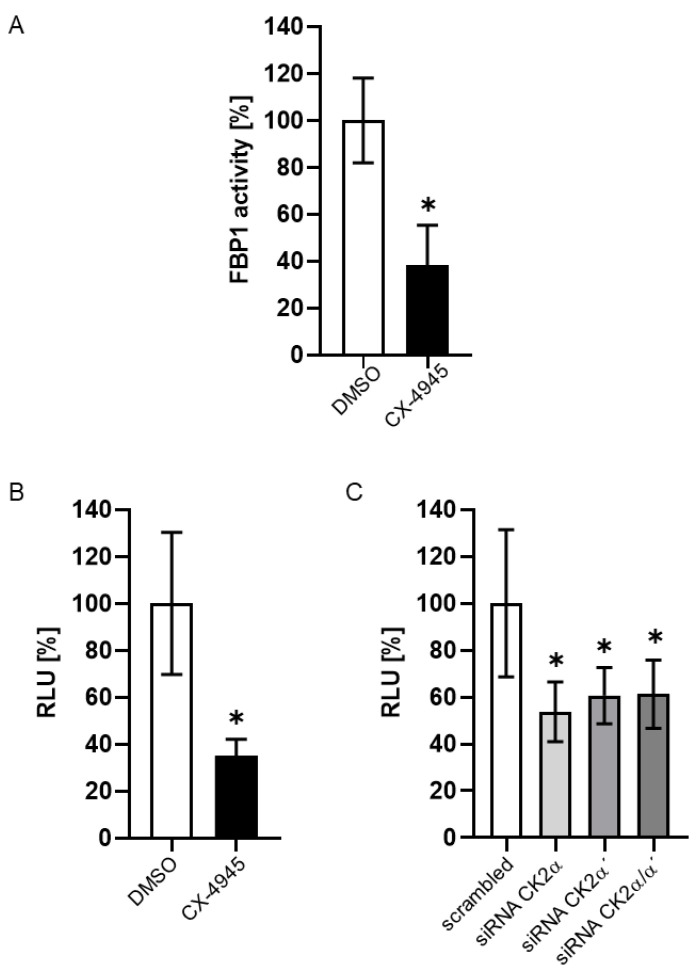
Impact of CK2 down-regulation on FBP1 activity in McA-RH7777 cells. (**A**) CK2 activity was inhibited by treatment with 10 µM CX-4945 and an equal volume of DMSO (for control). FBP1 enzymatic activity in McA-RH7777 cells was determined with a colorimetric assay as recommended by the manufacturer. FBP1 activity of cells treated with CX-4945 was set into reference to FBP1 activity of control cells (DMSO, 100%). The graph shows the result of at least three independent experiments. (**B**,**C**) CK2 activity was either inhibited by treatment with 10 µM CX-4945 and an equal volume of DMSO (for control) (**B**) or CK2 expression was down-regulated by transfection with siRNA against the catalytic subunits CK2α or CK2α´ (single or combined) or a scrambled siRNA for control (**C**). Cells were cultivated under conditions promoting gluconeogenesis. Extracellular glucose was determined in the cell culture supernatant using the GlucoseGlo^TM^ assay (Promega, Mannheim, Germany) according to the manual of the producer. Extracellular glucose of the treated cells was set in reference to the amount of extracellular glucose of the DMSO or the scrambled control (100%). The graphs show the result of at least three independent experiments. * Statistical significance was accepted as *p* < 0.05.

Next, we assessed the catalytic activity of FBP1 by means of a colorimetric assay. We treated McA-RH7777 cells with the CK2 inhibitor CX-4945 or the solvent control DMSO and analyzed the FBP1 activity. As shown in Figure 4A, we found a reduction in the FBP1 activity to 40% in the presence of CX-4945 compared to the solvent control (set 100%). Thus, these results are in accordance with the reduction in the expression of the FBP1 mRNA shown above. FBP1 is a key enzyme in gluconeogenesis. Therefore, we additionally determined the amount of glucose released from the cells after CK2 inhibition. As expected, we found markedly lower glucose concentrations in the supernatants of cells exposed to CX-4945 (Figure 4B) and in supernatants of cells treated with siRNAs for CK2α and/or CK2α´ (Figure 4C). All data presented here show that CK2 inhibition or down-regulation of CK2 subunits by siRNA technology resulted in a decrease in FBP1 enzyme activity and finally in a reduced secretion of glucose from liver cells.

**Figure 5:**
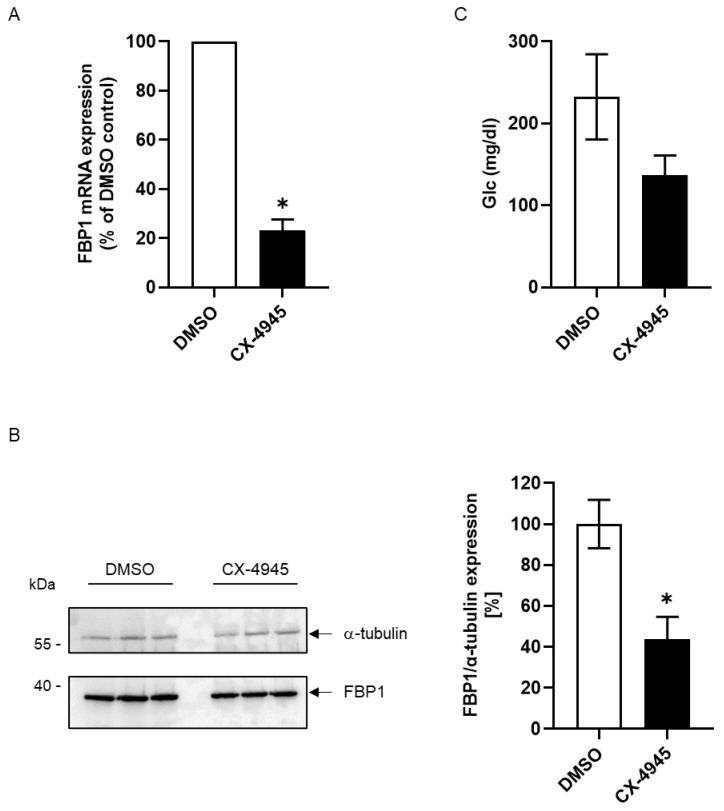
Impact of CX-4945 treatment of mice on FBP1 expression in liver and blood sugar level. Mice were treated for 3 days with CX-4945 or an equal volume of DMSO for control. Thereafter, they were sacrificed and liver and blood were retrieved and processed. (**A**) RNA was extracted from liver tissue, reverse transcribed into cDNA. cDNA was subjected to a qRT-PCR analysis using FBP1-specific primers and actin primers for housekeeping control. The graph shows the normalized value for control (DMSO)-treated and CX-4945-treated animals. (**B**) An amount of 5 µg each of liver tissue from three different mice was loaded on a 12.5% gel, separated, blotted onto a PVDF membrane and subjected to an immunoblot analysis with FBP1-specific antibody and α-tubulin-specific antibody for loading control. The bar graph shows the relative FBP1 expression after normalizing to tubulin. (**C**) Blood glucose concentration was determined in the serum of DMSO- and CX-4945-treated mice with the glucose dehydrogenase method. The graphs show the result of at least three independent experiments. * Statistical significance was accepted as *p* < 0.05.

In contrast to findings in whole organisms, experiments with cell lines lack the interaction with other cell types. In particular for the regulation of glucose homeostasis, the communication between different organs and different cells is vital. In order to find out whether the down-regulation of CK2 activity has also an influence on the FBP1 expression in organisms, we treated mice over three days with CX-4945 or an equal volume of DMSO as control. We determined the FBP1 mRNA expression in liver tissue by qRT-PCR as well as the protein expression by immunoblot analysis. Results are shown in Figure 5A,B.

We observed a significant reduction in FBP1 mRNA in mice treated with CX-4945 to 20% of that found in control animals. Additionally, the expression of FBP1 protein is strongly reduced to approximately 40%. Since FBP1 is among the key enzymes of the gluconeogenic pathway, we asked whether the treatment with CX-4945 has also an impact of the glucose level in blood. We determined the blood glucose of treated and control mice with the glucose dehydrogenase method. Figure 5C demonstrates that the blood glucose level in mice treated with CX-4945 was drastically reduced from 230 mg/dL to 140 mg/dL. Thus, even in living organisms CK2 inhibition by treatment with CX-4945 has an impact on FBP1 mRNA and protein expression and moreover, on glucose homeostasis.

## 3. Discussion

We identified a new factor which is involved in the regulation of glucose homeostasis by protein kinase CK2 by using a qRT-PCR assay with 84 diabetes-associated genes, namely fructose-1,6-bisphosphatase (FBP1). After inhibition of the CK2 kinase activity with the established CK2 inhibitor CX-4945 or a down-regulation of CK2α and CK2α’ expression by specific siRNAs, for the first time, we noticed a decrease in the gene expression as well as a down-regulation of the phosphatase activity of FBP1 in pancreatic β-cells as well as in hepatocellular carcinoma cells, which finally led to a reduction in glucose secretion. By using an animal model where we treated mice with the CK2 inhibitor CX-4945, we could demonstrate these observations also in living organisms. Formerly, we observed that inhibition of CK2 resulted also in an increase in the production and secretion of insulin from pancreatic β-cells [19,20,22,35,36]. Thus, inhibition of CK2 promotes diverse processes by which the glucose level is reduced. Remarkably, Marselli et al. [37] found that altered CK2 activity and expression were associated with an impaired β-cell function and insulin resistance. Individuals with type 2 diabetes mellitus showed a ~1.2-fold increase in CK2α gene (CSNK2A1) expression compared to nondiabetic individuals. Moreover, the CK2α amount in the serum of diabetic patients was higher than that in healthy donors, suggesting that CK2 is up-regulated in obese type 2 diabetes mellitus subjects, although these data did not reach statistical significance [38].

FBP1 plays a central role in gluconeogenesis, which becomes obvious in a rare inherited disorder, namely fructose-1,6-bisphosphatase deficiency (OMIM database entry #229700) where the patients suffer from severe hypoglycemia and metabolic acidosis on fasting [39]. FBP1 is the main isoform in the liver [24] but, there are many observations that FBP1 has also a broad expression in other tissues such as intestine, prostate, adrenal gland and pancreas [40,41]. The fact that there is no simultaneous detection of PEPCK in the pancreatic tissue led to the hypothesis that gluconeogenesis is started from trioses which are introduced in this pathway after phosphoenolpyruvate. Moreover, the authors have the evidence that FBP1 in pancreas plays a potential key role in glucose sensing and integration of metabolic signals to induce oscillatory insulin secretion [41,42,43]. They observed that inhibition of FBP1 activity in islet β-cells led to a significant increase in their glucose utilization and an enhanced glucose stimulated insulin secretion in vitro. On the other hand, up-regulation of FBP1 in pancreatic islet β-cells contributed to insulin secretory dysfunction. In light of our new data, it is tempting to speculate that CK2 influences the insulin secretion from β-cells not only by targeting PDX1 but, also by regulating the FBP1 expression. FBP1 is up-regulated in islets and pancreatic β-cells when exposed to a high-fat diet [44,45]. Up-regulation of FBP1 triggers gluconeogenesis and may also inhibit glycolysis, because it counteracts the reaction catalyzed by phosphofructokinase. Thus, FBP1 up-regulation will aggravate the hyperglycemic situation in type 2 diabetes mellitus. Therefore, it might be a potential target for the treatment of type 2 diabetes mellitus [46,47].

Early observations demonstrated that CK2 has a role in modulating activities of enzymes directly involved in carbohydrate storage and metabolism [6]. Among the first CK2 substrates described was glycogen synthase [48] whose activity during growth is controlled by an orchestrated action of PP2A and CK2 [49]. Phosphoglucose isomerase is a glycolytic enzyme, which is phosphorylated at serine 185 by CK2 [50]. A decrease in CK2 led to an increase in phosphoglucose isomerase activity.

Our present results suggest a transcriptional control of the expression of FBP1 by CK2. Detailed analysis of the FBP1 promoter revealed that USF1/USF2 bind to E-box motifs, SP1 and SP3 to GC- box elements and NF-κB to additional sequences in the FBP1 promoter [51]. USF1/USF2 are members of the helix-loop-helix leucine zipper family of proteins [52]. We have recently shown that USF1 but not USF2 is phosphorylated by CK2. Inhibition of CK2 kinase activity enhanced the dimerization of both proteins [53]. Furthermore, we found that the PDX1 promoter is repressed by USF1. Increasing glucose concentrations abrogated the repression of the PDX1 promoter by USF1. Inhibition of the kinase activity of CK2 identified CK2 as a negative regulator of USF1 regulated transcription of PDX1 [21].

NF-κB is also a substrate for CK2 [54,55,56,57]. siRNA induced down-regulation of CK2 decreases NF-κB nuclear localization, DNA binding and transcriptional activity [58]. The transcription factor SP1 is also a substrate for CK2 and the CK2 mediated phosphorylation of SP1 decreases its DNA binding activity and thereby transcription factor activity [59]. How this network of CK2-regulated transcription factors might regulate FBP1 transcription remains to be elucidated.

Our present study adds FBP1 as a new player to the network of CK2 modulated factors in the regulation of glucose homeostasis. We have not only shown in established cell lines but also in living animals that CK2 affects this important enzyme of the gluconeogenic pathway. However, whether this enzyme is the only target of the CK2-regulated network awaits further experiments.

## 4. Materials and Methods

### 4.1. Cell Culture and Treatment of Cells

The adherent rat insulinoma β cell line INS-1 832/13 [25] was cultivated in 100 mm cell culture dishes at 37 °C and 5% CO_2_ in a humidified atmosphere. The RPMI 1640 medium (GIBCO^TM^ by Thermo Fisher Scientific GmbH, Dreieich, Germany) supplemented with 10% fetal bovine serum, 1 mM sodium pyruvate and 50 µM mercaptoethanol served as the culture medium. In order to ensure the glucose responsiveness characteristic of the INS-1 832/13 cells, all experiments were carried out only with cells whose passage number was below 60.

The rat hepatoma cell line McA-RH7777 (ATCC^®^ CRL-1601™, LGC Standards GmbH–Germany Office, Wesel, Germany) was cultivated with DMEM (GIBCO^TM^ by Thermo Fisher Scientific GmbH, Dreieich, Germany) supplemented with 10% fetal bovine serum, and 1 mM sodium pyruvate under the same conditions.

One day before treatment with the CK2 inhibitor CX-4945 (SelleckChem, Munich, Germany), cells were seeded in such a way that a confluence of about 60–70% could be expected on the day of treatment. An appropriate volume of a 10 mM stock solution of CX-4945 dissolved in DMSO was added to the cells to get a final concentration of 2.5, 5 or 10 µM. An equal volume of the solvent DMSO was added to control cells. At 24 h after application, cells were harvested with a cell scraper and sedimented by centrifugation (7 min, 4 °C, 400× *g*). Cells were washed twice with cold phosphate-buffered saline (PBS) and then, subjected to the subsequent analyses.

### 4.2. Animals and Treatment

BALB/c mice were daily treated by intraperitoneal injection of CX-4945 (1.5 mg/kg dissolved in DMSO/PBS) for 3 days. Thereafter, the mice were sacrificed by cervical dislocation and the tissues samples of liver and blood were collected for additional analyses. The animal experiments are approved by the local governmental animal care committee (Landesamt für Verbraucherschutz, Abteilung C Lebensmittel- und Veterinärwesen, Saarbrücken, Germany) and conducted in accordance with the European legislation on protection of animals (Guide line 2010/63/EU) and the NIH Guidelines for the Care and Use of Laboratory Animals.

### 4.3. Transfection of McA-RH7777 Cells with siRNA

For transfection the following day, the cells were seeded in such a way that on the day of treatment, there was an approximate confluence of 70–80%. siRNAs against the catalytic subunits CK2α or CK2α′ were from DharmaconTM (On-TARGET-plus SMARTpool siRNA L-096197-02-0005 and L-092756-02-0005, Horizon Discovery Biosciences Limited, Cambridge, UK), scrambled nonsense siRNA was used as control (D-001810-10-05 ON-TARGETplus Nontargeting Pool, Horizon Discovery Biosciences Limited, Cambridge, UK).

The transfection was carried out according to the protocol of the manufacturer in a 6 well plate. The corresponding amount of siRNA was mixed in a serum-free medium with the transfection reagent DharmaFECT 1 (Horizon Discovery Biosciences Limited, Cambridge, UK) and incubated for 5 min at room temperature. For the transfection we used 100 nM siRNA per well in 200 μL serum-free medium and 10 μL DharmaFECT 1 in 200 μL serum-free medium. The mixture of both was then added to the cell layer and cells were cultivated over 72 h. Cells were harvested as described above.

### 4.4. RNA Extraction and Quantitative Real-Time PCR (qRT-PCR)

Cells were harvested by trypsinizing and were subsequently centrifugated (7 min, 4 °C, 400× *g*). RNA was extracted from treated and control cells using the QIAzol lysis reagent (Qiagen, Hilden, Germany) according to the manufacturer’s instructions. For qRT-PCR, RNA was subsequently washed with 75% ethanol. After drying, RNA was resuspended in RNAse-free water and the concentration was determined using a NanoDrop UV-Vis Spectrophotometer. mRNA was reverse transcribed to cDNA with a qScriber cDNA Synthesis Kit (HighQu, Kraichtal, Germany) according to the manufacturer’s protocol.

For the isolation of RNA from mouse liver tissue, Qiazol lysis reagent was immediately added after removal of the organ. The tissue was broken down using a homogenizer and RNA was isolated according to the instructions of the manufacturer (Qiagen, Hilden, Germany).

For amplification of the FBP1 cDNA, we applied the ORA SEE qPCR Green ROX L Mix Kit (HighQu, Kraichtal, Germany). We strictly followed the recommendations of the producer. Briefly, 100 ng of total RNA per reaction were reverse transcribed and analyzed in a one-step reaction using the primer combinations shown in Table 1. Actin served as an endogenous control for mRNA detection. The data acquisition was carried out with the MiniOpticon™ Real-Time PCR Detection System and the CFX Manager™ Software (CFX Maestro 2.2 (version 5.2.008.0222), Bio-Rad, Feldkirchen, Germany). The data were quantitatively analyzed using the ΔΔCt method.

### 4.5. qRT PCR Microarray

The Rat Diabetes RT² Profiler PCR Array (PARN-023ZA) from Qiagen (Hilden, Germany) profiles the expression of 84 genes related to the onset, development, and progression of diabetes. With the help of corresponding primer sets and the reverse transcriptase of the RT^2^ First Strand Kit (Qiagen, Hilden, Germany), the reverse transcription was carried out according to the manufacturer´s instructions, whereby 1 μg of the previously isolated total RNA from INS-1 832/13 cells, either treated with DMSO for control or with the CK2 inhibitor CX-4945 was converted into cDNA. In addition, contamination with genomic DNA was eliminated with an appropriate buffer contained in the kit. The reverse transcription reaction took place for 15 min at 42 °C before the enzyme was inactivated at 95 °C after 5 min of incubation. The amplification of the previously obtained cDNA was optically tracked using the DNA-intercalating fluorescent dye SYBR Green I contained in the RT² SYBR Green master mix (Qiagen, Hilden, Germany). The qRT-PCR was carried out in a 96 well format and read out in the Mx3000P qPCR system of Agilent Technologies (Santa Clara, CA, USA). Subsequently, the evaluation was carried out according to the ΔΔCT method.

### 4.6. Protein Extraction and Immunoblot Analysis

After harvesting the cells by scraping and sedimentation by centrifugation (7 min, 4 °C, 400× *g*), the cell pellet was lysed with the double to triple volume of RIPA buffer (50 mM Tris/HCl, pH 8.0, 150 mM NaCl, 0.5% sodium desoxycholate, 1% Triton X-100, 0.1% sodium dodecylsulfate) supplemented with both, the protease inhibitor cocktail Complete™ (1:25) and the phosphatase inhibitor PhosSTOP™ (1:10) (both from Roche Diagnostics, Mannheim, Germany). The cell suspension was incubated on ice for 20 min. After lysis cell debris was removed by centrifugation (20 min, 4 °C, 12,500× *g*). The protein content was determined according to a modified Bradford method (BioRad, Munich, Germany).

For protein extraction of mouse liver, the tissue sample was frozen in liquid nitrogen and crushed in a mortar. The broken tissue was resuspended in 3 volumes RIPA buffer with the protease and phosphatase inhibitor cocktail. The suspension was incubated for 45 min on ice. The tissue extract was centrifuged as above. The supernatant was used for the determination of the protein content.

SDS polyacrylamide gel electrophoresis and Western Blot analysis was performed essentially as described [53]. The PVDF membranes were incubated with the primary antibodies in TBS supplemented with 0.1% Tween20 (TBS-T) and 5% BSA for 1 h at room temperature or overnight at 4 °C. We used polyclonal rabbit sera against CK2α and CK2α´ [54] and a mouse monoclonal anti-CK2β antibody (sc-46666, Santa Cruz Biotechnologies, Heidelberg, Germany). PARP was detected with a polyclonal rabbit antibody (#9542, Cell Signaling Technology, Frankfurt a. M., Germany) and FBP1 with a monoclonal rabbit antibody (D2T7F Cell Signaling Technology, Frankfurt a. M., Germany). For the verification of equal loading we used a mouse monoclonal α-tubulin antibody (clone DM1A, T9026, Sigma-Aldrich, München, Germany). Membranes were washed twice with TBS-T buffer and incubated with the horseradish peroxidase (HRP)-conjugated secondary antibodies anti-rabbit-IgG (ab205718, Abcam, Cambridge, UK) or anti-mouse IgG (115-035-146, Dianova, Hamburg, Germany) for 1 h at room temperature. The expression of the proteins was visualized by enhanced chemoluminescence using the SuperSignal™ West Pico PLUS Chemiluminescent Substrate (Thermo Fisher Scientific GmbH, Dreieich, Germany).

### 4.7. In Vitro Phosphorylation

The enzymatic activity of protein kinase CK2 in the cell extract was analyzed in vitro by the incorporation of radioactively labelled phosphate from [^32^P]γATP into a synthetic substrate peptide with the amino acid sequence RRRDDDSDDD. By measuring the Čerenkov radiation, the amount of phosphate incorporated was determined. For this purpose, 10 μg protein of the total cell extract was made up with CK2 kinase buffer (50 mM Tris-HCl, pH 7.5, 150 mM NaCl, 5 mM MgCl_2_, 1 mM DTT) to a total volume 20 μL. After the addition of 30 μL radioactive CK2 reaction mix (25 mM Tris-HCl, pH 8.5, 150 mM NaCl, 5 mM MgCl_2_, 1 mM DTT, 50 μM ATP, 0.19 mM synthetic peptide, 20 μCi [^32^P]γATP/1 mL (Hartmann Analytic, Braunschweig, Germany)), the mixture was incubated for 5 min at 37 °C. The reaction was stopped on ice and 40 μL of the mix was transferred onto a Whatman P81 ion exchange filter paper (Sigma-Aldrich, Munich, Germany), which binds the peptide substrate. The filter paper was washed three times with 85 mM phosphoric acid and once with ethanol, for 5 min each. The filter paper was dried under red light and the emitted Čerenkov radiation was measured in a scintillation counter.

### 4.8. Determination of Fructose-1,6-Bisphosphatase Activity

The activity of fructose-1,6-bisphosphatase in cells was measured with a colorimetric assay (ab273329, Abcam, Cambridge, UK) according to the protocol of the manufacturer. For determination of the activity 50 µg of protein extract of McA-RH7777 cells was used, which were treated either with 10 µM CX-4945 or an equal volume of DMSO. The resulting absorbance (E450 nm) was measured in kinetic mode. FBP1 activity was calculated from the linear range of the resulting curve as suggested by the manufacturer and expressed as relative amount, setting the activity of DMSO-treated control cells to 100%.

### 4.9. Detection of Extracellular Glucose

To study glucose production through gluconeogenesis, McA-RH7777 cells were cultivated under appropriate conditions. Cells were treated with CK2 inhibitor or transfected with siRNA as described above. The night before the assay, DMEM high-glucose medium (4.5 g glucose/L) was replaced by low-glucose medium (1 g glucose/L). The next day, the medium was withdrawn and cells were washed twice with Krebs-Ringer buffer KRB (120 mM NaCl, 5 mM KCl, 2 mM CaCl_2_, and 1 mM MgSO_4_) to remove any residual glucose. Cells were incubated for another 2 h with KRB. Gluconeogenesis was induced by the addition of 10 mM lactate and 10 µM forskolin in KRB. After one hour incubation at 37 °C, supernatants were collected and secreted glucose was determined with the bioluminescent Glucose-Glo^TM^ Assay from Promega (Mannheim, Germany) according to the recommendations of the manufacturer. Luminescence was recorded in an Infinite M200 Pro TECAN Reader (Sigma-Aldrich, München, Germany) using the luminescence mode.

### 4.10. Determination of Blood Glucose

Blood samples were taken from the *Vena cava* from mice treated either with DMSO or with CX-4945. Blood was collected in tubes coated with EDTA. Samples were centrifuged for 10 min at 14,000× *g* to remove the blood cells. The concentration of glucose in serum was determined with the glucose dehydrogenase method (LT-SYS Glucose Dehydrogenase LT-GL 0022, Labor + Technik Eberhard Lehmann GmbH, Berlin, Germany) according to the instructions of the manufacturer.

### 4.11. Statistical Analysis

Data are presented as the mean ±  SD for each group. Differences between control groups and treated groups were analyzed using the Student *t* test or one-way ANOVA. *p*  <  0.05 was considered statistically significant. Data shown are representative of at least three independent experiments.

## Figures and Tables

**Table 1:** Primer pairs applied in qRT-PCR.

Target	Direction	Sequence
FBP1	forward	5′-CATCTGGAAAGCTGCGGCTGCTGTACG -3′
FBP1	reverse	5′-AGGGACGGCCTTGATTTGGCTTTGTCC -3′
Actin	forward	5′-CCT CTG AAC CCT AAG GCC AAC CGT GA -3′
Actin	reverse	5′-GGA CAA CAC AGC CTG GAT GGC TAC G -3′

## Data Availability

Not applicable.
